# Screening and brief interventions for hazardous and harmful alcohol use in probation services: a cluster randomised controlled trial protocol

**DOI:** 10.1186/1471-2458-9-418

**Published:** 2009-11-18

**Authors:** Dorothy Newbury-Birch, Martin Bland, Paul Cassidy, Simon Coulton, Paolo Deluca, Colin Drummond, Eilish Gilvarry, Christine Godfrey, Nick Heather, Eileen Kaner, Judy Myles, Adenekan Oyefeso, Steve Parrott, Katherine Perryman, Tom Phillips, Don Shenker, Jonathan Shepherd

**Affiliations:** 1 Institute of Health and Society, Newcastle University, Newcastle, UK; 2 Department of Health Sciences, University of York, York, UK; 3 Teams Family Practice, Gateshead, UK; 4 Centre for Health Service Studies, University of Kent, Canterbury, UK; 5 Section of Alcohol research, Institute of Psychiatry, King's College, London, UK; 6 Northern Regional Drug and Alcohol Services, Newcastle, UK; 7 School of Psychology and Sports Science, Northumbria University, UK; 8 Division of Mental Health, St George's University of London, UK; 9 Humber Mental Health and Teaching NHS Trust, Willerby, UK; 10 Alcohol Concern, London, UK; 11 Violence Research Group, Cardiff University, Cardiff, UK

## Abstract

**Background:**

A large number of randomised controlled trials in health settings have consistently reported positive effects of brief intervention in terms of reductions in alcohol use. However, although alcohol misuse is common amongst offenders, there is limited evidence of alcohol brief interventions in the criminal justice field. This factorial pragmatic cluster randomised controlled trial with Offender Managers (OMs) as the unit of randomisation will evaluate the effectiveness and cost-effectiveness of different models of screening to identify hazardous and harmful drinkers in probation and different intensities of brief intervention to reduce excessive drinking in probation clients.

**Methods and design:**

Ninety-six OMs from 9 probation areas across 3 English regions (the North East Region (n = 4) and London and the South East Regions (n = 5)) will be recruited. OMs will be randomly allocated to one of three intervention conditions: a client information leaflet control condition (n = 32 OMs); 5-minute simple structured advice (n = 32 OMs) and 20-minute brief lifestyle counselling delivered by an Alcohol Health Worker (n = 32 OMs). Randomisation will be stratified by probation area. To test the relative effectiveness of different screening methods all OMs will be randomised to either the Modified Single Item Screening Questionnaire (M-SASQ) or the Fast Alcohol Screening Test (FAST). There will be a minimum of 480 clients recruited into the trial. There will be an intention to treat analysis of study outcomes at 6 and 12 months post intervention. Analysis will include client measures (screening result, weekly alcohol consumption, alcohol-related problems, re-offending, public service use and quality of life) and implementation measures from OMs (the extent of screening and brief intervention beyond the minimum recruitment threshold will provide data on acceptability and feasibility of different models of brief intervention). We will also examine the practitioner and organisational factors associated with successful implementation.

**Discussion:**

The trial will evaluate the impact of screening and brief alcohol intervention in routine probation work and therefore its findings will be highly relevant to probation teams and thus the criminal justice system in the UK.

Ethical approval was given by Northern & Yorkshire REC

**Trial Registration number:**

ISRCTN 19160244

## Background

In 2008 in the UK it was estimated that there were approximately 950,000 incidents of alcohol-related violence in the previous year [1]. Alcohol is a factor in nearly half of all violent crimes (defined as assaults, robbery and snatch thefts in which the victim considered the perpetrator to be 'under the influence' of alcohol) [1]. High rates of alcohol and other substance misuse has been found amongst offenders within the prison system in the UK [2-5]. There is therefore strong evidence of an association between alcohol and offending behaviour [4,6,7]. However it is difficult to identify the precise causal relationships between alcohol misuse and offending behaviour [8-10].

The majority of alcohol-related problems in the general population are not due to individuals with alcohol dependence, but to a much larger group of hazardous and harmful drinkers [11]. In the UK, hazardous and harmful drinkers outnumber dependent drinkers by a ratio of 7:1 [11]. Thus, it is clear that the greatest impact in reducing alcohol-related problems at a population level can be made by reducing alcohol consumption in hazardous and harmful drinkers, rather than by focusing on the most extreme cases of alcohol dependence; this is known as the preventive paradox [12]. This present study will help to provide better information into this.

Screening and brief alcohol intervention is an example of secondary prevention [13]; it aims to identify hazardous and harmful drinking at an early stage, before people are consciously aware of the potentially harmful effects of their drinking or are alcohol help seeking, and then deliver advice or counselling to help reduce consumption levels.

### Current evidence on screening and brief interventions

There is a strong evidence-base supporting the effectiveness of brief intervention (BI) in reducing alcohol consumption in adults who are not seeking treatment for alcohol-related problems in a range of health settings. Numerous systematic reviews and meta-analyses have reported beneficial outcomes of brief intervention, compared to control conditions, in terms of reductions in hazardous and harmful drinking [14-23]. However there is a lack of data on the impact of screening and brief interventions within the criminal justice system. Although some studies show promise [24,25], methodological limitations include non-randomised trials and small sample sizes, limiting the conclusions which can be made. A study of offenders in a magistrates' court who had been sentenced for a violent offence committed while intoxicated with alcohol examined re-offending, injury rates and subjects' alcohol-related risk and harm status [25]. Male participants were followed up at three and twelve months post-sentence. Although there was an initial reduction in alcohol use at the 3-month follow up this was not found to be significant [25]. However, there was a significantly lower rate of injury in the offenders who received the intervention compared to those who had not [25].

In summary, there is a need for more research evaluating the impact of screening and brief interventions in the criminal justice system. It is also necessary to ensure that research trials are located in areas with sufficient diversity to be generalisable. In addition, there are key gaps in the evidence-base regarding the cost-effectiveness of brief interventions, the efficiency of screening activity and the optimal methods of brief intervention. Although there have been reported cost-savings in the US in primary care four years post brief alcohol intervention [26] this evidence is lacking in the criminal justice system.

During late 2007 we conducted a pilot study to evaluate the most feasible criminal justice setting to conduct a randomised controlled trial of brief interventions. The main findings of the pilot were:

• The prevalence of individuals with an alcohol use disorder (AUD) in the criminal justice setting is 3 times greater than in the general population.

• The consent rate for study participation, of those eligible, was highest in the probation setting.

• The majority of offenders who were positive on the AUDIT questionnaire [27] reported not feeling coerced to take part in any further research.

• The recruitment, consent and willingness to participate rates were higher in the probation setting.

Of the four settings (probation, prison, police stations and Youth Offending Teams) probation was found to be the most feasible setting to undertake the trial.

## Aim of the study

To evaluate the most efficient and acceptable screening tool to detect hazardous and harmful drinkers in typical probation offices and to evaluate the staff impact and cost effectiveness of different models of brief intervention aimed at reducing excessive drinking in this setting.

## Objectives

The objectives of the trial are:

• To conduct a pragmatic multicentre cluster randomised controlled trial of screening and brief intervention for hazardous and harmful drinkers in typical probation settings in three English regions.

• To identify the most efficient methods for alcohol screening in typical probation settings.

• To compare the effectiveness and cost effectiveness of different models of brief intervention with an information leaflet in offenders with hazardous, harmful and dependent alcohol consumption.

• To assess the implementation of different screening and brief intervention approaches by Offender Managers (OMs) in typical probation practice.

• To identify attitudinal, practical, skill, resource, and reinforcing factors that predict successful implementation of screening and brief intervention in probation settings.

• To assess the relative impact of the different models of screening and brief intervention on uptake of alcohol services, including an alcohol helpline.

• To assess the relative impact of the three intervention strategies on reoffending.

## Methods/Design

### Setting

Ninety-six OMs from 9 probation areas across 3 English regions (the North East Region (n = 4) and London and the South East Regions (n = 5)) will be recruited. All probation offices delivering general probation services in the three regions that do not have current routine screening and brief intervention facilities will be eligible to participate. The study catchment area enables broad population coverage and Randomisation procedures will ensure that offices cover a range of urban and rural areas, socially deprived and affluent communities, traditional communities with relatively stable populations and more urbanized fluid populations, and culturally mixed populations.

### Design

The trial will adopt a 2 × 3 nested factorial design encompassing screening method (the Fast Alcohol Screening Test (FAST) [28] or a modified Single Alcohol Screening Questionnaire (M-SASQ)) [29]) and brief intervention intensity (Client information leaflet (CIL); Brief advice (BA); Brief lifestyle counselling (BLC)). The main advantages of utilising a factorial approach are twofold. First each of the elements (screening tool and intervention) can be analysed independently with sufficient power to make meaningful interpretation of relative effectiveness. Second, the method enables meaningful interpretations of the relative effectiveness of any screening tool and intervention modality.

The 96 recruited OMs will be randomly assigned to 1 of 2 screening tools (48 OMs using each tool) and 1 of 3 intervention conditions (32 OMs in each condition) as shown in Table 1. Since we expect that there may be a difference in uptake of screening between intervention conditions, we expect the recruitment to take varying periods of time in the different conditions.

**Table 1:** SIPS CJS trial design

	Intervention allocation				Total
		**PIL**	**BA**	**BLC**	
	
		OMs = 16	OMs = 16	OMs = 16	OMs = 48
**Screening allocation**	**FAST**	Participants = 80	Participants = 80	Participants = 80	Participants = 240
	
	**M-SASQ**	OMs = 16	OMs = 16	OMs = 16	OMs = 48
		Participants = 80	Participants = 80	Participants = 80	Participants = 240

		OMs = 32	OMs = 32	OMs = 32	OMs = 96
**Total**		Participants = 160	Participants = 160	Participants = 160	Participants = 480

The trial will incorporate cluster randomisation of OMs to avoid the risk of contamination. OMs trained to deliver brief advice become compromised in their ability to deliver alternative versions of such care; thus it is not practical for individual OMs to deliver both control and intervention conditions in this trial. Therefore OMs will consistently deliver one particular type of brief intervention.

### Study Hypotheses

• Brief lifestyle counselling by an Alcohol Health Worker (AHW) is more effective and cost effective than simple structured advice conducted by OMs in a typical probation setting.

• Brief lifestyle counselling and simple structured advice are more effective and cost effective than a Client Information Leaflet (CIL).

• Access to referral to an AHW results in greater screening and intervention activity than training of probation staff in screening and brief intervention alone.

• Attitudinal, practical skill, resource, and reinforcing factors predict screening and intervention activity.

### Probation Office recruitment

Contact with probation offices will initially be made via regional Chief Probation Officers and regional alcohol leads for probation. Thereafter visits to probation offices will be arranged to enable research staff to explain the trial protocol, secure staff consent to participate and to organize the probation office-based training.

### Inclusion Criteria

#### Probation offices

All probation offices that have not already instigated screening and brief intervention systems.

#### Offenders

Any offender with a positive screening result on FAST or M-SASQ, who is alert and orientated aged 18 or over, resident within 20 miles and able to speak, read and write English sufficiently well to complete study questionnaires.

### Exclusion Criteria

Offenders will not be eligible if they are already seeking treatment for an AUD or are taking part in another study of alcohol interventions, if they are severely injured or suffering with a serious mental health problem, or who are grossly intoxicated. Finally offenders with no fixed abode will be excluded from the study.

### Randomisation

Randomisation will be conducted using a secure remote randomisation service. Blocks containing six possible allocations will be generated for each of the permutations of screening method (FAST vs M-SASQ) and intervention (CIL vs BA vs BLC). OMs will be randomly assigned to each allocation en masse at the start of the trial. Randomisation will be stratified by geographical area.

### Screening

In order to test the relative effectiveness of different screening methods in identifying hazardous and harmful drinkers we intend to conduct a cluster randomised comparison of two validated screening tools. A modified version of the M-SASQ which asks "How often do you have X or more standard drinks on one occasion?" where X = 6 for women and 8 for men, with monthly, or weekly, or daily or almost daily considered a positive screen [29]. We tested the M-SASQ in a pilot study and established a sensitivity of 91.8 and specificity of 70.8 when compared to the gold standard Alcohol Use Disorders Identification Test (AUDIT) [27]. FAST [28] is a universal screening tool that has high sensitivity and specificity in health settings ([28]. All screening tools have been modified to include a visual guide to interpreting a 'standard drink'. (A standard drink = 8 mg of pure ethanol = 1 UK Unit of alcohol).

All offenders who are attending probation and potentially eligible will be informed of the study and asked to consent to be screened. They will be screened using the instrument available to the OM. Participants will be informed of the outcome of the screening.

In all conditions, the research team will support participating OMs in implementing screening systems tailored to the needs of the probation office.

### Consent

Consent to participate will be obtained in a three- stage process. OMs will give every offender a copy of the Client Information Leaflet (CIL) to take away to read before the next appointment. At the next appointment, OMs will ask the client if they have read the leaflet and whether they have any questions. OMs will then establish verbal consent to confirm eligibility to take part, collect some basic demographic information and to be screened for an alcohol use disorder. Those who then are positive on the screening tool will have the study explained to them verbally by OMs and in writing (using the Client Information Leaflet). Written informed consent will be obtained by OMs. This will include permission to give the client's data and contact details to the research team, and provide the research team with access to the clients Police National Computer (PNC) and probation records, and to participate in follow up after 6 and 12 months. The research team will then contact the participant within two weeks to thank him/her for taking part in the study.

## Interventions

### Client information leaflet

OMs randomised to the control condition, participating staff will be trained to screen eligible clients for hazardous or harmful drinking. Clients who screen positive and provide consent to participate in the study will complete the baseline questionnaire and then will be provided with a client information leaflet (CIL) http://www.sips.iop.kcl.ac.uk. The CIL to be used in this trial will be the Department of Health's "How much is too much; Drinking and You" leaflet [30]. This information booklet contains useful information about alcohol and includes the Drinkline telephone number where the client can access further information including treatment options for alcohol problems. Details of local alcohol services will also be attached to the back cover of the CIL.

### Brief advice condition

OMs randomised to deliver Brief Advice, will be trained to screen eligible offenders for hazardous or harmful drinking. Offenders who screen positive and provide consent to participate in the study will complete the baseline questionnaire and will then receive up to five minutes of simple structured brief advice from trained OMs using the SIPS Brief Advice tool "Brief advice about alcohol risk" which has been developed for the purpose of the SIPS programme. It is based on the "How much is too much?" Simple Structured Advice intervention tool, developed as part of the UK version of the Drink-Less Brief Intervention programme [31] from a prototype used as part of a World Health Organisation collaborative study on alcohol screening and brief intervention [32]. Offenders in this condition will also receive a Client Information Leaflet (CIL) from the OM at the end of the brief advice.

### Brief lifestyle counselling

OMs randomised to deliver Brief Lifestyle Counseling, will be trained to screen eligible offenders for hazardous or harmful drinking. Offenders who screen positive and provide consent to participate in the study will complete the baseline questionnaire and will then receive up to five minutes of simple structured brief advice from trained OMS using the SIPS Brief Advice tool "Brief advice about alcohol risk". Offenders in this condition will also receive a Client Information Leaflet (CIL) at the end of the brief advice. OMs will also be trained to refer offenders to an AHW, by making an appointment usually the following day or as soon as possible after probation attendance. The AHW will be experienced in carrying out alcohol assessment and brief interventions. The AHW will deliver a 20 minute brief lifestyle counseling intervention to offenders who attend the appointment at the probation office, using the SIPS Brief Lifestyle Counselling (BLC) Tool which has been developed for the purpose of the SIPS programme. It is based on the "How much is too much?" Extended Brief Intervention tool developed as part of the UK version of the Drink-Less BI programme [31] from a prototype used as part of a World Health Organisation collaborative study on alcohol screening and brief intervention [32].

All intervention tools and protocols are available from the SIPS study website http://www.sips.iop.kcl.ac.uk.

### Training and support

All OMs participating in the trial will be trained to implement alcohol screening and brief intervention according to the trial protocol. The aim of the training is to provide some background information about alcohol related harm, to give an overview of the study protocol, to familiarise staff with the screening tools, structure and scoring procedures, and to inform staff about the procedure for implementing screening and brief intervention in their site. Given the cluster design of the trial, staff will only be introduced to the screening tool they have been randomly allocated to. The training is individualised according to the implementation procedure agreed with the local collaborator and senior colleagues in probation.

A substantial element of the training will involve the understanding and familiarisation of alcohol units to ensure that the OMs are fully aware of the alcohol content of different alcoholic drinks so they are fully able to complete the screening tools accurately. Moreover, as the screening tools refer to standard drinks rather than Units when assessing consumption, the training ensures that staff are aware that a standard drink is one Unit and that they are able to convert different drinks into the number of standard drinks. Visual representations of standard drinks as well as several examples of people's drinking patterns are used to allow trainees to practice calculating in the alcohol content of each drink and to add up the number of standard drinks consumed in order to identify positive cases.

### Training of staff to deliver brief advice

Participating staff selected to deliver brief advice in probation randomised to either the Brief Advice condition or the Brief Lifestyle Condition will receive a one hour training session on how to deliver five minutes of brief advice according to the protocol.

The aim of the training is to provide OMs with the skills necessary to effectively deliver brief advice about alcohol risk to offenders attending the probation office in which the work. The training was developed by the SIPS team to be delivered by an AHW. The AHWs the SIPS team are experienced practitioners in the field of alcohol interventions. They contributed to the development of the training package and have been fully trained to deliver the training to practitioners.

The standard training package is based on a PowerPoint presentation with scripts to standardise delivery. The training sessions will be adapted for use in the different experimental conditions in which BA is being delivered.

The session will be presented to small groups of OMs who are encouraged to interact with the trainer, ask questions and comment on the content. This will be followed by an interactive role play session in which the AHW demonstrates the intervention, and then each OM will have an opportunity to practice with a co-worker, observed by the trainer who will provide feedback and encouragement. Training sessions will be delivered to groups of 1-6 practitioners with 1-3 being the typical group size.

### Training of staff to deliver brief lifestyle counselling

AHWs will be recruited to deliver the BLC within the participating probation offices. Staff recruited will, as a minimum, possess; a relevant professional qualification, a diploma in drug and/or alcohol studies or equivalent, 5 years post-qualifying experience with a minimum 2 years in an alcohol or drug speciality, prior knowledge and understanding of psychological interventions including motivational interviewing.

All AHW's will receive formal training and supervision from the point of recruitment. Training will be based upon the previous work of Rollnick et al [33] in addition to experiences from an earlier trial of screening and brief interventions [34]. The training will comprise of four main elements; orientation to the relevant OM, taught workshops, tape recorded simulated consultations with trained actors and ongoing clinical supervision provided by experienced senior clinicians.

The simulated consultations will be recorded and rated by three independent clinical assessors. The AHW will be assessed for adherence to the BLC protocol in addition to their behaviour and skills using the Behaviour Change Counselling Index (BECCI) [35]. Assessors will submit BECCI ratings, comments and supervision points for each consultation. This information will support clinical supervision and training until the AHW reaches a required standard of practice agreed by an independent clinical assessor.

## Outcome Measures

Participating OMs will be surveyed before and after training in each condition of the study to assess attitudinal factors and factors influencing implementation of screening and brief intervention procedures.

Attitudes will be assessed via the shortened Alcohol and Alcohol Problems Perception Questionnaire (SAAPPQ) [36]. A list of all OMs that can deliver alcohol screening and brief intervention in each study site will be compiled. A self-administered SAAPPQ will be distributed to participating staff on three occasions: pre- and post-training and post-study. SAAPPQ has five subscales covering role adequacy, role legitimacy, self-esteem, motivation, and work satisfaction. Role adequacy and role legitimacy are concerned with role security, i.e., how individuals perceive the adequacy of their skills and knowledge in relation to problem drinkers and how appropriate it is for them to work with such offenders. The subscales relating to self esteem, motivation and work satisfaction, are concerned with worker's therapeutic commitment, i.e., the extent to which they seek to engage drinkers in treatment and the extent that they find the work rewarding on both a professional or personal level [37].

In addition to the SAAPPQ, the post-training and post-study questionnaires will contain a number of semi-structured and open questions developed to elicit information on staff attitudes towards alcohol screening and brief intervention; previous experience of delivering alcohol screening and brief intervention; readiness to undertake these activities; the training needed to conduct screening and brief intervention; the suitability of each site to provide SBI; and potential barriers to effective implementation.

Factors relevant to implementation of screening and brief intervention have been found to be divided into *predisposing*, *enabling *and *reinforcing *factors [38]. Predisposing factors relate to Offender Managers' willingness to implement screening and brief intervention. Enabling factors are the skills and resources needed to implement screening and brief intervention. Reinforcing factors are visible results, feedback from peers and patients and other factors that encourage continuation of screening and brief intervention.

In this study we will modify the above [37] to survey Offender Managers before and after training and compare the above factors between different implementation models.

### System measures

The research team will identify the total number of offenders aged over 18 years who attended appointments with the Offender Managers in the trial during the recruitment period, the total number of offenders screened, the number screening positive and the number receiving an alcohol intervention in each of the 3 implementation models. This will allow calculation of the overall screening rate, the screen conversion rate (proportion of positive screens) and the intervention rate in the different settings. We will also compare these measures and the FAST versus M-SASQ screening tools.

Offenders' re-attendances at probation offices over the 6 and 12 month follow-up periods will be assessed using computerised records and compared with attendances by participating offenders in the 6 and 12 months before entry into the study. The sustainability of the screening and intervention approaches will be assessed by examining the extent to which screening and intervention activity continues after the end of the formal study recruitment period.

### Client measures

#### Baseline

Immediately before receiving the initial CIL and/or brief advice intervention, participants will be invited by the Offender Manager to provide contact details and complete the Extended AUDIT [27], Euroqol (EQ-5D) [39], a short service use questionnaire (S-SUQ) [40] and modified Readiness Ruler [41]. Participants in the extended intervention will complete the baseline at the same stage as those in other groups.

AUDIT is normally used as a screening test for alcohol use disorders [27]. However in this context the AUDIT will be used as a means of establishing the severity of alcohol use disorders at baseline, in a way that is least intrusive to naturalistic aim of the trial in the probation setting and as a means of measuring the adequacy of matching between the intervention groups at baseline. The AUDIT contains 10 items to measure alcohol consumption, alcohol problems and dependence over, in this case, the previous 6 months, and the sum of the item scores provides a measure of severity which has been used in several previous studies, allowing comparability with other primary care samples [11]. We felt that the use of more elaborate baseline alcohol consumption measures would interfere with the naturalistic aims of the study and possibly would contribute a form of intervention in itself, so introducing bias into the evaluation of the interventions by reducing the difference between trial interventions. In addition, participants will complete the EQ-5D as a brief 5-item measure of quality of life [39]. Use of health, social criminal justice services and wider societal costs will be measured via a shortened version of Service Use Questionnaire [40] which allows estimation of health care and wider social costs for health economic analysis in the six months prior to intervention. A modified Readiness Ruler [41] containing a zero to ten scale of the extent to which participants think about their drinking as a problem or have addressed this issue will assess participants' motivational state regarding changing their drinking behaviour.

#### Follow-up

At 6 and 12 months after intervention, all participants will be contacted via telephone, post or email as preferred by research staff that will be blind to their intervention condition. Participants will be offered telephone, postal or face-to-face follow-up as preferred. Researchers will administer the shorter Alcohol Use Disorders Identification Test (Extended-AUDIT) [42]. Alcohol-related problems will be assessed via the brief Alcohol Problems Questionnaire (APQ) [43]. We will also re-administer an extended version of the Service Use Questionnaire SUQ [40], EQ-5D [39] and the modified Readiness Ruler [40]. Participant satisfaction with the advice/help received during the intervention will be assessed using a modified version of the Patient Satisfaction Questionnaire (short form) at 12 months [44].

At follow-up, each participants will also be asked if, and how often, they made use of the Drink-Line telephone number.

### Financial incentives

We propose to incentivise research participation in one of two ways dependent on individual offices choice. They will either receive via payments of £1,000 to each probation site geographical area subject to successful offender recruitment or the participating OMS will receive £20.00 of high street vouchers for each eligible case recruited.

### Participant incentives

Participants will receive a £10 retail voucher after completing the baseline research interview and a £10 voucher for completing each of the 6 and 12 month research follow-up interviews.

### Economic evaluation

The economic component of the study comprises a cost-effectiveness and cost-utility analysis. The study aims to identify, quantify and value resources related to alcohol screening and intervention by OMs in probation and the subsequent use of health, social care, and criminal justice services by offenders following each type of intervention. Resources utilised in the identification and brief intervention delivery or control condition will be recorded by OMs involved on an ongoing basis. This will allow the calculation of costs related to implementation of different models of screening and brief intervention. Local costs will be used to calculate the costs of the interventions, which will include staff costs, premises costs and costs of leaflets and other consumables. In addition, specific training costs for staff will be calculated, in terms of staff time, premises costs and the cost of training materials.

Offenders' self-reported use of health, social care and criminal justice services will be identified retrospectively using a short form of the service use questionnaire previously used to evaluate costs associated with interventions for alcohol use disorders [40] and applying a common set of national unit cost estimates. The economic analysis will calculate the incremental cost-effectiveness of the control condition with the AHW condition under study, using measures of clinical outcome and quality of life, EQ-5D [45] responses at baseline and at 6 and 12 months follow up. The use of EQ-5D enables the estimation of Quality Adjusted Life Years. Data will be bootstrapped to account for the expected skewness evident in economic cost data [46]. The analysis will include the construction of cost-effectiveness acceptability curves to illustrate the probability that the brief intervention is more cost-effective than usual care, based on different monetary values being attached to QALYs. The use of QALYs follows the recommendations of NICE and enables the value for money afforded by treatment to be compared to a range of other health care interventions. Furthermore, combination of the economic cost data and outcome data with offender data collected in the trial will enable a secondary analysis of various offender characteristics that may influence the cost-effectiveness of the intervention.

### Cost effectiveness of crime

The economic analysis currently is designed to consider cost effectiveness in terms of incremental cost per QALY. However, one of the key outcomes of interest to criminal justice providers is re-offending. We will therefore use these additional data to undertake subsidiary cost-effective analysis and consider whether the choice of principal outcome measure alters the recommended decisions. The two outcomes chosen will be number of offences and time to failure and the analysis will be conducted on the sample participants with both QALY and CJS data.

### Sample size calculation

The sample size calculation is designed to account primarily for intervention level outcomes. Powering the study in this way will also account statistically for appropriate outcomes for screening approach and screening method. The primary outcome for this study is the proportion of offenders who are consuming alcohol within recommended levels at 6 and 12 month follow-up. Recent meta-analysis [19] suggests that the difference between brief intervention and control is of the order 13%, 5% reduction in the control group and 18% in the brief intervention group. In order to detect a difference of this magnitude at the 5% significance level with 80% power, for a 2-sided test, requires 109 offenders in each of the 3 groups, a total of 327. Our experience with other multi-centre randomised controlled trials of interventions for alcohol use disorders suggests that with assiduous follow-up the potential loss to follow-up across groups is of the order 25%. Taking this loss into accounts inflates the sample required to 145 in each group, a total of 435 offenders.

The proposed study involves a cluster design and requires a statistical adjustment to account for any potential cluster effect. The literature is unclear regarding an appropriate estimation of an intra-class correlation coefficient in this population. Our experience leads us to assume the ICC should be similar to primary care correlation coefficients, 0.04. Assuming a cluster size of the order 3 offenders this inflates the sample size calculation by a factor of 1.1 requiring 160 offenders in each group, a total of 480, with an expectation that at least 365 will be followed up at 12 months.

### Planned analysis

As the study is pragmatic in design, the planned analysis will be by intention to treat. The primary outcome is dichotomous in nature, drinking within or above recommended levels, and will be analysed with logistic regression adjusting for all known prognostic factors; data will be presented as odds ratios and their corresponding confidence intervals. Secondary analyses will be undertaken using the appropriate method for the outcomes, controlling where appropriate for intake values and other known prognostic variables using analysis of covariance.

Due to the nested factorial nature of the study, we will use multi-level modelling to explore potential interactions between each of the levels nested within the trial (screening method and intervention). Offender manager and offender factors will be utilised as part of regression model to explore possible prognostic factors that impact on outcome. Interaction analysis will explore any possible interactions between site and offender characteristics and outcome.

The efficacy of interventions will be explored using a per protocol approach to the analysis. A sub-group, of those who received their allocated treatment will be utilised for this analysis.

### Ethical and Research Governance Approval

The study has been granted ethical approval by multi-centres research ethics committee (MREC reference number: 08/H0903/2).

### Project timescales

The trial duration is 30 months and it commenced in May 2008

## Discussion

Whilst there is evidence to support screening and brief interventions in the health setting there is at yet very little in the criminal justice field. The proposed trial will provide evidence that will go towards informing probation practices in England, the UK and outside the UK on screening and brief advice. In addition the trial will consider the effectiveness and cost-effectiveness of screening and brief intervention at reducing alcohol consumption and its related problems, including re-offending whilst providing important information about implementation issues which will have an effect on future implementation by probation.

## Competing interests

The authors declare that they have no competing interests.

## Authors' contributions

All of the authors contributed to the design and development of this trial protocol. CD is the Chief Investigator of SIPS and EK is Deputy Chief Investigator. Expertise on criminal justice aspects of the research was provided by DNB, AO and JS. Expertise on clinical aspects of the research was provided for primary care by PC and JM, for nursing practice by TP and for psychiatry CD and EG. Clinical supervision of the AHWs provided by CD and EG. Statistical input was provided by SC and MB. Health economics input was provided by CG and SP. Trial management, conduct and delivery expertise was provided by PD, DNB and KP. Alcohol and policy expertise was provided by AO and DS. Brief intervention expertise was provided by CD, EK, NH and JS. DNB wrote the first draft of the paper and all authors contributed to successive drafts. All authors read and approved the final manuscript.

## Pre-publication history

The pre-publication history for this paper can be accessed here:

http://www.biomedcentral.com/1471-2458/9/418/prepub

## References

[B1] Kershaw C Nicholas S Walker A Crime in England and Wales 2007/2008 2008 London: Home Office Statistical Bulletin

[B2] Office for National Statistics Substance misuse among prisoners in England and Wales London 1999

[B3] Fazel S Bains P Doll H Substance abuse and dependence in prisoners: a systematic review Addiction 2006 101 2 181 191 10.1111/j.1360-0443.2006.01316.x 16445547

[B4] McMurran M Baldwin S Services for prisoners with alcohol-related problems: a survey of UK prisons Addiction 2006 84 9 1053 1058 10.1111/j.1360-0443.1989.tb00788.x 2790268

[B5] Shaw J Hunt I Flynn S Amos T Meehan J Robinson J Bickley H Parsons R McCann K Burns J The role of alcohol and drugs in homicides in England and Wales Addiction 2006 101 8 1117 1124 10.1111/j.1360-0443.2006.01483.x 16869841

[B6] Richardson A Budd T Young adults, alcohol, crime and disorder Criminal Behaviour and Mental Health 2003 13 1 5 16 10.1002/cbm.527 14624268

[B7] Ireland C Thommeny J The crime cocktail: licensed premises, alcohol and street offences Drug and Alcohol Review 1993 12 2 143 150 10.1080/09595239300185571 16818323

[B8] Collins J Drinking and Crime 1982 London: Tavistock

[B9] Pernanen K Alcohol and Human Violence 1991 New York: Guildford Press

[B10] Plant M Plant M Thornton C People and places: Some factors in the alcohol violence link Journal of Substance Use 2002 7 207 213 10.1080/14659890215690

[B11] Drummond C Oyefeso A Phillips T Cheeta S Deluca P Perryman K Winfield H Jenner J Cobain K Galea S Alcohol needs assessment research project (ANARP). The 2004 national needs assessment for England 2004 London: Department of Health and the National Treatment Agency

[B12] Kreitman N Alcohol consumption and the preventive paradox British Journal of Addictions 1986 81 353 363 10.1111/j.1360-0443.1986.tb00342.x 3461846

[B13] Winett RA A framework for health promotion and disease prevention programs American Psychologist 1995 50 5 341 350 10.1037/0003-066X.50.5.341 7762887

[B14] Agosti V The efficacy of treatments in reducing alcohol consumption: a meta-analysis International Journal of the Addictions 1995 30 8 1067 1077 7558483 10.3109/10826089509055828

[B15] Bien TH Miller WR Tonigan JS Brief interventions for alcohol problems: a review Addiction 1993 88 3 315 335 10.1111/j.1360-0443.1993.tb00820.x 8461850

[B16] Freemantle N Gill P Godfrey C Long A Richards C Sheldon T Song F Webb J Brief Interventions and Alcohol Use Effective Health Care Bulletin 1993 7 1 13 10.1136/qshc.2.4.267 PMC1055159 10132464

[B17] Wilk AI Jensen NM Havinghurst TC Meta-analysis of randomized control trials addressing brief interventions in heavy alcohol drinkers Journal of General Internal Medicine 1997 12 274 283 10.1007/s11606-006-5063-z 9159696 PMC1497107

[B18] Poikolainen K Effectiveness of brief interventions to reduce alcohol intake in primary health care populations: a meta-analysis Preventive Medicine 1999 28 503 509 10.1006/pmed.1999.0467 10329341

[B19] Moyer A Finney JW Swearingen CE Vergun P Brief interventions for alcohol problems: a meta-analytic review of controlled investigations in treatment-seeking and non-treatment-seeking populations Addiction 2002 97 3 279 292 10.1046/j.1360-0443.2002.00018.x 11964101

[B20] Ballesteros JA Duffy JC Querejeta I Arino J Gonzalez-Pinto A Efficacy of brief interventions for hazardous drinkers in primary care: systematic review and meta-analysis Alcoholism, Clinical & Experimental Research 2004 28 4 608 618 10.1097/01.alc.0000122106.84718.67 15100612

[B21] Bertholet N Daeppen J-B Wietlisbach V Fleming M Burnand B Brief alcohol intervention in primary care: systematic review and meta-analysis Archives of Internal Medicine 2005 165 986 995 10.1001/archinte.165.9.986 15883236

[B22] Whitlock EP Polen MR Green CA Orleans T Klein J Behavioral counseling interventions in primary care to reduce risky/harmful alcohol use by adults: a summary of the evidence for the US Preventive Services Task Force Annals of Internal Medicine 2004 140 557 568 15068985 10.7326/0003-4819-140-7-200404060-00017

[B23] Kaner E Beyer F Dickinson H Pienaar E Campbell F Schlesinger C Heather N Saunders J Burnand B Effectiveness of brief alcohol interventions in primary care populations Cochrane Database of Systematic Reviews 2007 2 CD004148 10.1002/14651858.CD004148.pub3 17443541

[B24] Hopkins M Sparrow P Sobering up: Arrest referral and brief intervention for alcohol users in the custody suite Criminology and Criminal Justice 2006 6 4 389 410 10.1177/1748895806068576

[B25] Watt K Shepherd J Newcombe R Drunk and dangerous: a randomised controlled trial of alcohol brief intervention for violent offenders Journal of Experimental Criminology 2008 4 1 19 10.1007/s11292-007-9048-7

[B26] Fleming MF Mundt MP French MT Manwell LB Stauffacher EA Barry KL Benefit-cost analysis of brief physician advice with problem drinkers in primary care settings Medical Care 2000 38 1 7 18 10.1097/00005650-200001000-00003 10630716

[B27] Saunders JB Aasland OG Babor TF De La Fuente JR Grant M Development of the Alcohol Use Disorders Identification Test (AUDIT): WHO Collaborative Project on Early Detection of Persons with Harmful Alcohol Consumption Addiction 1993 88 6 791 804 10.1111/j.1360-0443.1993.tb02093.x 8329970

[B28] Hodgson R Alwyn T John B Thom B Smith A The FAST alcohol screening test Alcohol & Alcoholism 2002 37 1 61 66 10.1093/alcalc/37.1.61 11825859

[B29] Canagasaby A Vinson DC Screening for hazardous or harmful drinking using one or two quantity-frequency questions Alcohol & Alcoholism 2005 40 3 208 213 10.1093/alcalc/agh156 15797883

[B30] Department of Health How Much is Too Much Crown Copyright 2007

[B31] McAvoy B Kaner E Haighton K Heather N Gilvarry E 'Drink-Less' - Marketing a brief intervention package in UK general practice Family Practice 1997 14 5 427 428

[B32] Centre for Drug and Alcohol Studies The Drink-Less Programme 1993 Australia: Department of Psychiatry, Sydney

[B33] Rollnick S Mason P Butler C Health Behaviour Change: A guide for practitioners 1999 Edinburgh: Churchill Livingstone

[B34] Drummond D James D Coulton S Parrott S Baxter J Ford D Godfrey C Lervy B Peters T Russell I The effectiveness and cost-effectiveness of screening and stepped-care interventions for alcohol use disorders in the primary care setting Final report to the Welsh Office for Research and Development 2003

[B35] Lane C The Behaviour Change Counselling Index (BECCI) Scale 2002 Cardiff: University of Wales

[B36] Anderson P Clement S The AAPPQ revisited: The measurement of general practitioners attitudes to alcohol problems British Journal of Addiction 1987 82 753 759 10.1111/j.1360-0443.1987.tb01542.x 3478065

[B37] Gorman DM Cartwright AKJ Implications of using the composite and short versions of the Alcohol and Alcohol Problems Perception Questionnaire (AAPPQ) British Journal of Addiction 1991 86 327 334 10.1111/j.1360-0443.1991.tb01786.x 2025696

[B38] Babor TE Higgins-Biddle J Dauser D Higgins P Burleson JA Alcohol screening and brief intervention in primary care settings: implementation models and predictors Journal of Studies on Alcohol 2005 66 3 361 368 16047525 10.15288/jsa.2005.66.361

[B39] Rabin R Charro F EQ-5D: a measure of health status from the euroqol group Annals of Medicine 2001 33 5 337 343 10.3109/07853890109002087 11491192

[B40] UKATT Research Team Cost effectiveness of treatment for alcohol problems: findings of the randomised UK alcohol treatment trial (UKATT) British Medical Journal 2005 331 7516 544 10.1136/bmj.331.7516.544 16150765 PMC1200587

[B41] Heather N Smailes D Cassidy P Development of a Readiness Ruler for use with alcohol brief interventions Drug and Alcohol Dependence 2008 98 235 240 10.1016/j.drugalcdep.2008.06.005 18639393

[B42] Bush K Kivlahan DR McDonell MB Fihn SD Bradley KA The AUDIT alcohol consumption questions (AUDIT-C): an effective brief screening test for problem drinking Archive of Internal Medicine 1998 158 1789 1795 10.1001/archinte.158.16.1789 9738608

[B43] Drummond DC The relationship between alcohol dependence and alcohol-related problems in a clinical population British Journal of Addiction 1990 85 357 366 10.1111/j.1360-0443.1990.tb00652.x 2334822

[B44] Ware J Snyder M Wright W Eds Development and validation of scales to measure patient satisfaction with medical care services 1976 Springfield VA: National Technical Information Service

[B45] The EuroQol Group "EuroQol - A new Facility for the Measurement of Health-Related Quality of Life" Health Policy 1990 16 3 199 208 10.1016/0168-8510(90)90421-9 10109801

[B46] National Institute for Clinical Excellence Guide to the methods of technology appraisal. Volume National primary care development team: Northern NPDT Centre 2004 London: National Institute for Clinical Excellence

